# Mutation Screening and Array Comparative Genomic Hybridization Using a 180K Oligonucleotide Array in VACTERL Association

**DOI:** 10.1371/journal.pone.0085313

**Published:** 2014-01-09

**Authors:** Johanna Winberg, Peter Gustavsson, Nikos Papadogiannakis, Ellika Sahlin, Frideborg Bradley, Edvard Nordenskjöld, Pär-Johan Svensson, Göran Annerén, Erik Iwarsson, Ann Nordgren, Agneta Nordenskjöld

**Affiliations:** 1 Department of Molecular Medicine and Surgery and Center for Molecular Medicine, Karolinska Institutet, Stockholm, Sweden; 2 Clinical Genetics Unit, Karolinska University Hospital, Stockholm, Sweden; 3 Department of Laboratory Medicine, Division of Pathology, Karolinska Institutet, Stockholm, Sweden; 4 Department of Women's and Children's Health and Center for Molecular Medicine, Karolinska Institutet, Stockholm, Sweden; 5 Pediatric Surgery, Astrid Lindgren Children's Hospital, Karolinska University Hospital, Stockholm, Sweden; 6 Department of Immunology, Genetics and Pathology, Uppsala University, Uppsala, Sweden; NIDCR/NIH, United States of America

## Abstract

In order to identify genetic causes of VACTERL association (V vertebral defects, A anorectal malformations, C cardiac defects, T tracheoesofageal fistula, E esophageal atresia, R renal anomalies, L limb deformities), we have collected DNA samples from 20 patients diagnosed with VACTERL or with a VACTERL-like phenotype as well as samples from 19 aborted fetal cases with VACTERL. To investigate the importance of gene dose alterations in the genetic etiology of VACTERL association we have performed a systematic analysis of this cohort using a 180K array comparative genomic hybridization (array-CGH) platform. In addition, to further clarify the significance of PCSK5, HOXD13 and CHD7 genes in the VACTERL phenotype, mutation screening has been performed. We identified pathogenic gene dose imbalances in two fetal cases; a hemizygous deletion of the FANCB gene and a (9;18)(p24;q12) unbalanced translocation. In addition, one pathogenic mutation in CHD7 was detected, while no apparent disease-causing mutations were found in HOXD13 or PCSK5. Our study shows that although large gene dose alterations do not seem to be a common cause in VACTERL association, array-CGH is still important in clinical diagnostics to identify disease cause in individual cases.

## Introduction

VACTERL association (OMIM #192350) is described as the non-random association of multiple congenital anomalies affecting the specific organ systems intended in its letters; V vertebral anomalies, A anal atresia, C cardiac defects, T tracheoesophageal fistula, E esophageal atresia, R renal anomalies and L limb defects. The diagnosis is based on clinical observation, with a minimum of three anomalies in the spectrum required for diagnosis and a reported prevalence of VACTERL association in infants in different studies between 1/10, 000 and 1/40, 000 [1].

At present, no common etiology of VACTERL association is known and a heterogeneous background to the phenotype is likely [1]. The included malformations develop at different time points during embryogenesis and the pathogenesis is thought to interfere with several different embryological processes [2]. In the majority of cases, VACTERL association occurs sporadically which has lead to suggestions of environmental causative factors [1], but the condition is also familial in rare cases [3], [4], [5] indicating the possibility of a genetic etiology [6]. Genetic aberrations reported in patients with VACTERL or a VACTERL-like phenotype include chromosome aberrations, microdeletions/-duplications and single gene mutations in individual cases [7], [8], [9], [10], [11], [12], [13], [14], [15], [16], [17]. Genetic counseling for families with VACTERL association based on current knowledge is unsatisfactory and studies exploring genetic and environmental factors are important to improve the situation as advances in pediatric surgery and intensive care have increased survival rates of children with multiple congenital anomalies.

The aim of the study was to determine the role of gene dose alterations in the etiology of VACTERL association through screening analysis using array-CGH in patients and fetal cases. We also wanted to clarify the role of PCSK5, HOXD13 and CHD7 variants in the VACTERL phenotype since PCSK5 and HOXD13 genetic variants have been reported in VACTERL patients [18], [19]. Screening of CHD7 was performed to investigate the presence of CHARGE patients within our VACTERL cohort, based on the known clinical overlap between the two conditions. In line with results of earlier studies, our study shows no common genetic cause in patients with VACTERL association and that disease-causing gene dose alterations are not commonly found in these patients. Further, the known clinical similarity between CHARGE and VACTERL association is strengthened by the finding of a pathogenic CHD7 mutation in a patient with a VACTERL-like phenotype.

## Materials and Methods

### Ethics statement

Patient participation was approved after obtaining written consent from the parents. Samples from fetal cases were obtained from a biobank to which the parents had agreed on storage of fetal tissue samples for research and clinical purposes. Ethical approval for the study was obtained from the regional ethics committee at Karolinska Institutet, Stockholm.

### Patients and fetal cases

Altogether, 39 patients and fetal cases with either VACTERL or a VACTERL-like phenotype were included in the study (Table 1 and Table S1). Twelve patients were recruited from the department of Pediatric Surgery at Astrid Lindgren Children's Hospital, Stockholm, and from the Swedish VACTERL Society. Clinical information was obtained from medical records and through self-reporting from parents. Further, we included 19 fetal cases retrieved from the archives of the Section for Perinatal Pathology at Karolinska University Hospital in Huddinge, Stockholm. Autopsy reports were reviewed together with an experienced perinatal pathologist (N.P.). In all but one case, the pregnancies had been terminated after routine fetal ultrasound had revealed multiple congenital anomalies. Finally, eight patients with esophageal atresia and a minimum of one additional malformation in the VACTERL spectrum were included.

**Table 1:** Summary of clinical phenotypes in patients and fetal cases with VACTERL association or a VACTERL-like phenotype.

Patients																				
	V1	V2	V3	V4	V5	V6	V7	V8	V9	V10	V11	V12	V13	V14	V15	V16	V17	V18	V19	V20
Sex	F	M	M	M	M	F	M	M	M	M	F	M	F	F	M	M	M	F	F	F
V		+	+		+		+	+	+		+	+		+						
A	+†	+	+	+	+	+	+		+			+	+				+	+		
C			+		+	+	+*		+	+	+				+	+			+	
TE			+	+		+		+		+	+		+	+	+	+	+	+	+	+
R	+	+		+	+		+	+			+		+			+		+		+
L	+				+	+	+					+		+						
Additional defects	+		+			+	+			+		+	+	+	+	+	+			

Fetal cases																			
	FC1	FC2	FC3	FC4	FC5	FC6	FC7	FC8	FC9	FC10	FC11	FC12	FC13	FC14	FC15	FC16	FC17	FC18	FC19
Sex	F	F	M	F	M	F	F	M	M	M	M	M	F	M	M	M	F	M	F
V							+	+			+				+				
A	+†		+	+			+		+	+	+	+	+		+	+	+	+	+
C	+	+	+	+	+	+	+	+	+	+	+		+	+	+	+	+		+
TE		+			+	+	+				+	+	+	+				+	+
R	+	+	+	+	+		+	+	+	+	+	+	+	+	+	+	+	+	+
L		+			+		+			+	+					+	+		
Additional defects	+	+	+	+	+	+	+	+	+	+	+	+	+	+	+	+	+	+	+

V vertebral defect, A anal atresia, C cardiac defect, TE esophageal atresia with/without tracheoesophageal fistula, R renal defect, L limb defect, †cloacal/cloacal-like malformation *atrial septal defect detected abroad at younger age while not detected at later investigation.

### DNA isolation

Genomic DNA was isolated from either peripheral blood or tissue samples according to standard procedures. For fetal cases, DNA was isolated from routinely preserved fresh frozen heart, liver, lung, or spleen tissue using the Gentra Puregene Blood Kit (QIAGEN Sciences, Maryland, USA) in combination with Proteinase K (Finnzymes, Espoo, Finland) with slight modifications to the manufacturer's protocol.

### Array-CGH

#### 180K oligonucleotide array

Screening for gene dose alterations was carried out using a 180K custom oligonucleotide microarray with whole genome coverage (Oxford Gene Technology) used in a clinical setting at the Department of Clinical Genetics, Karolinska University Hospital. Genomic DNA isolated from blood or tissue and sex-matched pooled reference DNA isolated from healthy controls (Promega, Madison, WI, USA) was used for analysis. Sample labelling (CGH labelling kit for oligo arrays, Enzo Life Sciences, Farmingdale, NY, USA), hybridization and slide washing (Oligo aCGH/ChIP-on-Chip Wash Buffer Kit, Agilent Technologies, Wilmington, DE, USA) was performed essentially according to the manufacturer's recommendations. Slide scanning was performed using a 3 µm-resolution microarray scanner and the Feature Extraction software v 10.7.3.1 (Agilent Technologies) was used for initial data analysis followed by analysis with the CytoSure Interpret Software v 3.3.2 (Oxford Gene Technology). For detection of copy number variants (CNVs), the standard probe cut off levels used in routine diagnostics at the Department of Clinical Genetics, Karolinska University Hospital (log2-ratios above 0.35 for duplications and below -0.65 for deletions) were applied. For an overall screening analysis, CNVs with a minimum of three consecutive probes with deviating log2-ratios were identified. After visual inspection, CNVs were compared to variants reported in the Database of Genomic Variants (DGV, http://projects.tcag.ca/variation/), DatabasE of Chromosomal Imbalance and Phenotype in Humans using Ensembl Resources (DECIPHER, https://decipher.sanger.ac.uk/) and the clinical database at the Department of Clinical Genetics, Karolinska University Hospital, comprising approximately 2000 analyzed patient samples. Detected CNVs overlapping CNVs reported in DGV or frequently observed in the clinical database were considered to be likely benign. Specifically, a visual inspection of probes located in PCSK5, HOXD13, the FANC genes (A, B, C, D2, E, F, G, I, L, M, N, O, P) and CHD7 was carried out. The data discussed in this section have been deposited in NCBI's Gene Expression Omnibus [20] and are accessible through GEO Series accession number GSE51958 (http://www.ncbi.nlm.nih.gov/geo/query/acc.cgi?acc=GSE51958).

#### Bacterial artificial chromosome (BAC) array

Early analysis using a 38K BAC array with whole genome coverage (Swegene DNA Microarray Resource Center, Department of Oncology, Lund University, Sweden) was performed as previously described in individual cases as specified in Table S2 [21].

### Multiplex Ligation-dependent Probe Amplification (MLPA)

Confirmation of the FANCB deletion detected with array-CGH was performed at Centogene GmbH, Rostock, Germany using the MLPA-P113-A2 kit from MRC Holland according to the manufacturer's recommendations.

### Polymerase chain reaction (PCR) and DNA sequencing

DNA isolated from blood or tissue was used for exon and exon/intron boundary amplification of the genes PCSK5 (exons 1–39), HOXD13 (exons 1–2) and CHD7 (exons 2–38) with PCR using standard procedures. Mutation screening in PCSK5 was performed in all patients while screening of HOXD13 and CHD7 was performed in a subset of patients (Table S2). All primer sequences and reaction conditions are available upon request. Sequencing reactions of amplified regions were carried out after a purification step using ExoSAP enzyme (ExoSAP-IT, GE Healthcare, GmbH, Germany) for 15 min at 37° followed by 15 min at 85°. The Big Dye Terminator cycle sequencing kit 3.1 (Applied Biosystems, Warrington, UK) was used for the sequencing reactions following the manufacturer's protocol and fragments were analysed on ABI 3130 and ABI 3730 genetic analysers (Applied Biosystems). SeqScape software V2.5 (Applied Biosystems) was used for sequence analysis. We used the Alamut v2.0 software (Interactive Biosoftware, http://www.interactivebiosoftware.com) to predict variant pathogenicity. Alignment of protein sequences for PCSK5 was performed using the UniProtKB database (http://www.uniprot.org/) and included sequences from 6 vertebrates.

### cDNA synthesis and sequencing

#### CHD7

As DNA from the parents was unavailable in patient V7, total RNA was isolated from peripheral blood of the patient (RNeasy, QIAGEN Sciences, USA) to investigate a potential effect of a detected variant on CHD7 splicing. cDNA synthesis was performed with the SuperScript® III First Strand Synthesis System kit (Invitrogen, Carlsbad, CA, USA) using oligo(dT)_20_-primers according to the manufacturer's protocol. Subsequent PCR using cDNA from patient V7 as well as a healthy control in parallel reactions using a forward primer located in exon 12 and a reverse primer located in exon 14 (Ensembl accession number ENSG00000171316) was carried out. Fragment sizes were compared using agarose gel electrophoresis. DNA sequencing of the amplified fragment was performed as described above.

### Protein-protein interaction database search

To test for protein-protein interactions between proteins encoded by genes in detected pathogenic CNVs or CNVs of unclear significance and VACTERL candidate genes, searches were performed in the PINA: Protein Interaction Network Analysis Platform database (http://cbg.garvan.unsw.edu.au/pina/) [22] (Table S3).

## Results

### Patients and fetal cases

Clinical data are shown in Table 1 and Table S1.

### Array-CGH

**Figure 1:**
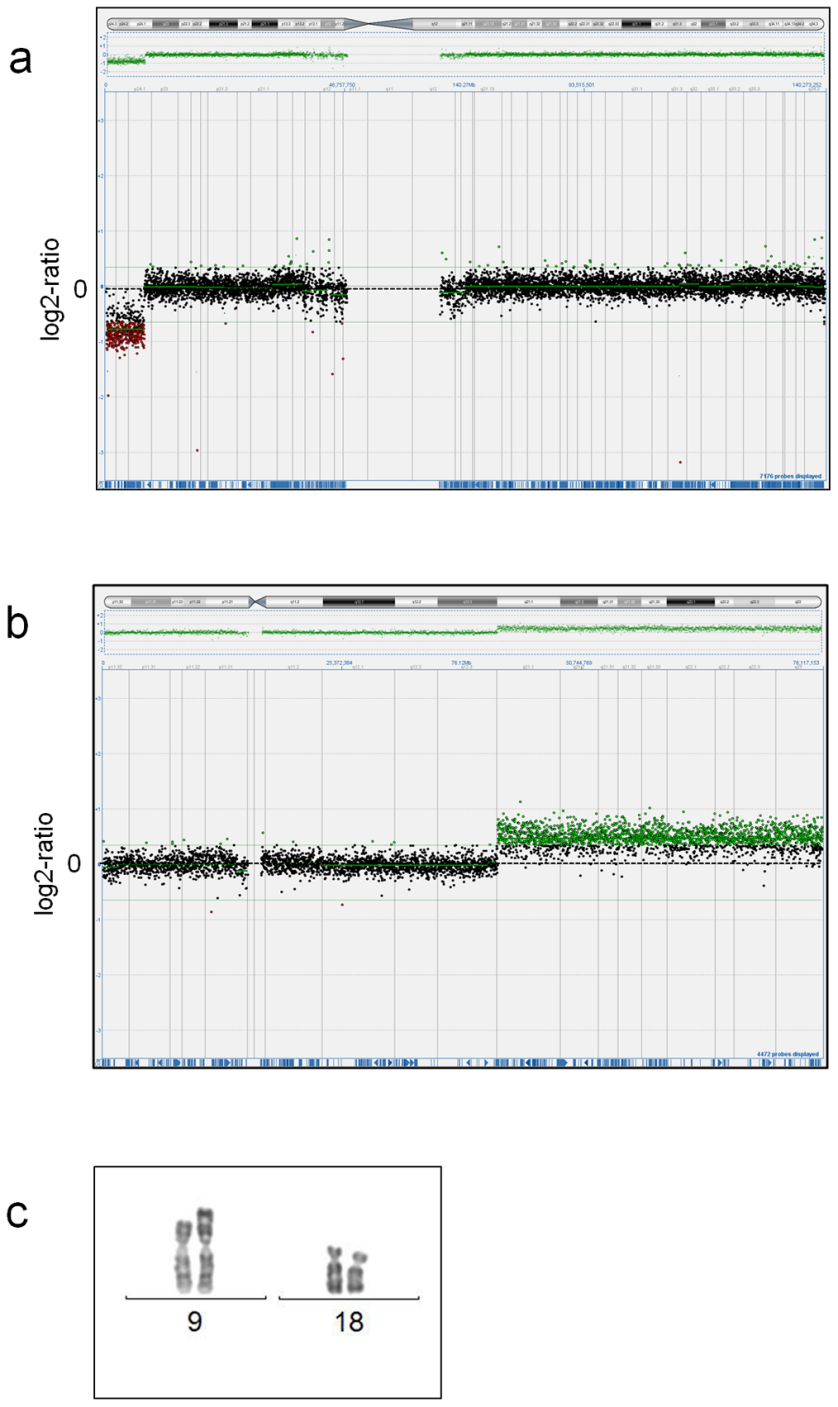
Results from array-CGH and karyotype analysis in fetal case FC14. For each probe, the log2-fluorescence signal ratio is displayed on the Y-axis and the chromosomal location on the X-axis. A 7.3 Mb-deletion on the p-arm of chromosome 9, del(9)(p24.3p24.1) (a) and a 34.3 Mb-duplication on the q-arm of chromosome 18, dup(18)(q12.3q23) (b) are shown. Both aberrations result from an unbalanced translocation involving chromosomes 9 and 18 (c).

**Figure 2:**
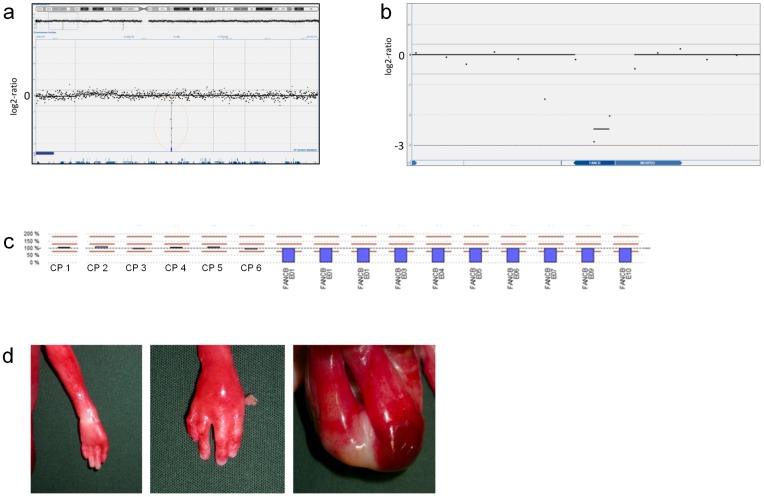
Results from array-CGH and MLPA analysis on genomic DNA from fetal case FC10. Upper images show a hemizygous deletion (mean log2-ratio -2.5) on Xp22.2 comprising two probes located within the *FANCB* gene consistent with a diagnosis of X-linked VACTERL (a, b). Results from MLPA analysis of *FANCB* in fetal case FC10 (c). Y-axis scale with 100% representing a normal copy number and values above and below representing duplications and deletions respectively (0% representing homo-/hemizygous deletion). Results shown as blue bars emanating from baseline (100%). Analysis results in FC10 showing six control probes with normal copy number and hemizygous deletions of all *FANCB* exons. Lower images show malformations identified in fetal case FC10: left thumb agenesis (left), right thumb hypoplasia (middle) and imperforate anus (right) (d).

Screening for gene dose imbalances revealed pathogenic aberrations in two fetal cases (Table 2). In case FC14 (Fig. 1a, b), a terminal 7.34 Mb deletion of 9p, del(9)(p24.3p24.1), was identified together with a terminal 34.3 Mb 18q-duplication, dup(18)(q12.3q23). The fetus showed a complex heart malformation, esophageal atresia, unilateral mild hydronephrosis and hydroureter in combination with dilatation of the 4^th^ cerebral ventricle. In this case, a fetal karyotype had been performed in early pregnancy that had revealed an unbalanced 9;18 translocation (Fig. 1c) inherited from the father who was a carrier of the balanced translocation, 46,XY,t(9;18)(p24.1;q12.3). In the male fetus FC10, with anal atresia, a complex Fallot-like heart malformation, horseshoe kidney, bilateral rudimentary thumbs and a malformed right ear with atresia of the external auditory canal, a hemizygous 0.1–0.4 Mb deletion of the FANCB gene was identified (Fig. 2a–d). MLPA for FANCB performed at the Centogene laboratory (http://www.centogene.com/) confirmed deletion of the complete FANCB gene in the fetus, a finding associated with the diagnosis of X-linked VACTERL (Fig. 2c). Carrier testing revealed that the mother was a healthy carrier. In addition, a 2.8 Mb-duplication on chromosome 16, dup(16)(p13.11p12.3), was detected in fetus FC10. The inheritance status of the duplication is not known since the mother is not a carrier, and paternal DNA is not available.

**Table 2:** Pathogenic gene dose imbalances identified using array-CGH.

Patient/fetal case	Detected variant	Affected genes	Inheritance	Clinical significance
FC10	del(X)(p22.2p22.2) 0.01–0.04 Mb	FANCB	maternal	pathogenic
	chrX:14785775-14797007		(X-linked recessive)	
FC14	del(9)(p24.3p24.1) 7.3 Mb	∼40 Ref Seq genes deleted	paternal	pathogenic
	chr9:194178-7534426		(father carrier of balanced 9;18 translocation)	
	dup(18)(q12.3q23) 34.3 Mb	∼160 Ref Seq genes duplicated	paternal	
	chr18:41777994-76116089		(father carrier of balanced 9;18 translocation)	

Genomic coordinates according to genomic build Mar. 2006 (NCBI36/hg18).

In total, we detected 26 additional gene dose alterations of unclear clinical significance that do not correspond to previously reported deletions or duplications in DGV or in the DECIPHER database (Table S4).

### DNA- and cDNA-sequencing

#### PCSK5

**Figure 3:**
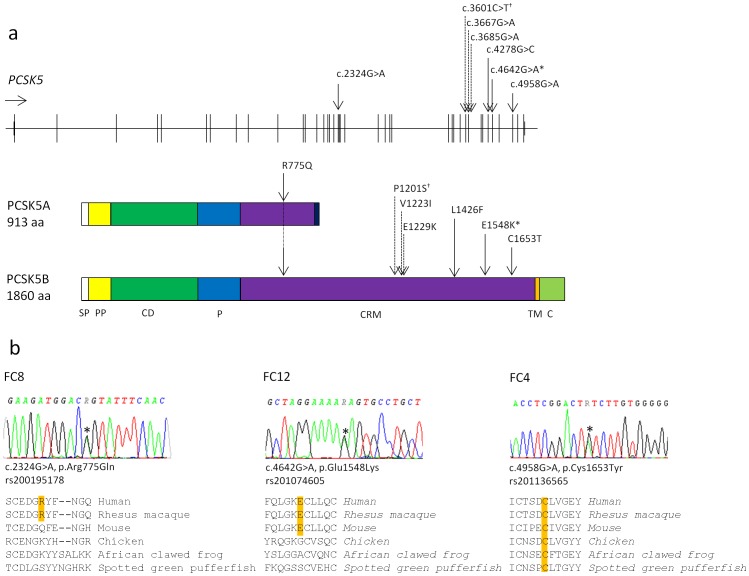
Results from DNA sequencing of the *PCSK5* gene and protein sequence alignment. a. Upper part showing exons of the *PCSK5* gene (ENSG00000099139) with location of detected sequence variants indicated by black arrows (our study) and crosshatched arrows (Szumska et al.)[18]. Star indicates variant detected in one fetal case in our study and in two patients by Szumska et al. (c.4642G>A, p.Glu1548Lys). Lower part of figure showing the two isoforms of PCSK5 with protein domains, based on image from Seidah et al. [35]. Prolonged arrow indicating the amino acid change p.Arg775Gln in both PCSK5 protein isoforms. Dagger indicates variant that was reported by Szumska et al. and was later reported as a non-synonymous coding SNP (rs114508164, P1201S). SP signal peptide, PP propeptide, CD catalytic domain, P P-domain, CRM cysteine-rich motif, TM transmembrane domain, C cytosolic tail. b. Results from *PCSK5* DNA sequencing show heterozygous missense variants (*) in fetal cases FC8, FC12 and FC4. For each detected variant, the corresponding protein sequence alignment of 6 vertebrate PCSK5B sequences is shown beneath, revealing conservation of Cys-1653 in all 6 species (mammals, birds, amphibians and fish), Glu-1548 in mammals and Arg-775 only in primates.

DNA-sequencing identified three heterozygous non-synonymous missense variants; one predicted to be damaging (c.4958G>A, p.Cys1653Tyr) and two predicted to be benign (c.2324G>A, p.Arg775Gln and c.4642G>A, p.Glu1548Lys), that were not detected in 95 healthy blood donors (Fig. 3a, Table 3). The p.Cys1653Tyr variant, predicted to be damaging, was detected in fetal case FC4 who showed anal atresia, a ventricular septal defect (VSD), unilateral renal agenesis, a hypoplastic 12^th^ rib pair and some dysmorphic facial features. In addition, a heterozygous non-synonymous missense variant in exon 32 was identified (c.4278G>C, p.Leu1426Phe) that was found in 1/95 healthy blood donors (Table 3). All the PCSK5 variants were identified in fetal cases without access to parental samples. All detected variants were recently reported in dbSNP, while only two (rs201136565 (p.Cys1653Tyr) and rs150021157 (p.Leu1426Phe)) were reported in the Exome Variant Server (Aug, 2013) (Table 3).

**Table 3:** *PCSK5* variants and corresponding phenotypes in fetal cases with VACTERL association.

Patient/ fetal case	Detected variant	V	A	C	T	E	R	L	Other birth defects	Inheritance	Presence in controls	GT freq. (dbSNP)	
FC4	c.4958G>A	x	x	x			x		x	na	0/95	rs201136565	
	p.Cys1653Tyr											A/G 0.3%	
FC8	c.2324G>A	x		x			x		x	na	0/95	rs200195178	
	p.Arg775Gln											no frequency data	
FC12	c.4642G>A		x		x	x	x		x	na	0/95	rs201074605	
	p.Glu1548Lys											A/G 1.1%	
FC19	c.4278G>C		x	x	x	x	x		x	na	1/95	rs150021157	
	p.Leu1426Phe											C/G 2.8%	
RC25^1^	p.Glu1229Lys	x†	x	x	x	x			x	maternal	-	-	
	(p.Glu1256Lys)												
RC37^1^	p.Glu1548Lys	x†*	x	x			x	x	x	ns	1/228	rs201074605	
	(p.Glu1575Lys)											A/G 1.1%	
goa13^1^	p.Glu1548Lys	x†		x	x	x		x	x	paternal	1/228	rs201074605	
	(p.Glu1575Lys)											A/G 1.1%	
RC15^1^	p.Pro1201Ser	x†*						x	x	maternal^2^	-	rs114508164	
	(p.Pro1228Ser)											no frequency data	
RC30^1^	Val1223Ile	x†					x		x	maternal	-	-	
	(Val1250Ile)												

*PCSK5* variants detected in this study and corresponding phenotypes presented together with *PCSK5* variants and phenotypes reported by Szumska et al [18]. Aa-residue numbering according to UniProtKB PCSK5 accession nr Q92824 (aa-numbering used by Szumska et al. shown beneath in parentheses), GT freq. genotype frequency, - not present, na not analysed, *ns* not stated, *Currarino syndrome diagnosis (*MNX1* status not reported), †sacral agenesis, ^1^patients reported by Szumska et al., ^2^present also in two healthy sisters with heterozygous and homozygous variants respectively.

#### HOXD13

A paternally inherited heterozygous 9 bp-deletion in a polyalanine region of HOXD13 was identified in patient V4 (Table 4), resulting in a loss of three alanine residues on protein level, consistent with a reported polymorphism (http://www.uniprot.org/).

**Table 4:** Detected variants in *HOXD13* and *CHD7*.

Patient	Detected variant	Inheritance	Clinical significance
V4	*HOXD13*	paternal	likely benign
	c.183_191del		
	p.Ala182_Ala184del		
V15	*CHD7*	*de novo*	pathogenic
	c.3202-1 G>C		
V7	*CHD7*	na	likely benign
	c.3202-5 T>C		

Variants detected after DNA sequencing of *HOXD13* and *CHD7*.

#### CHD7

**Figure 4:**
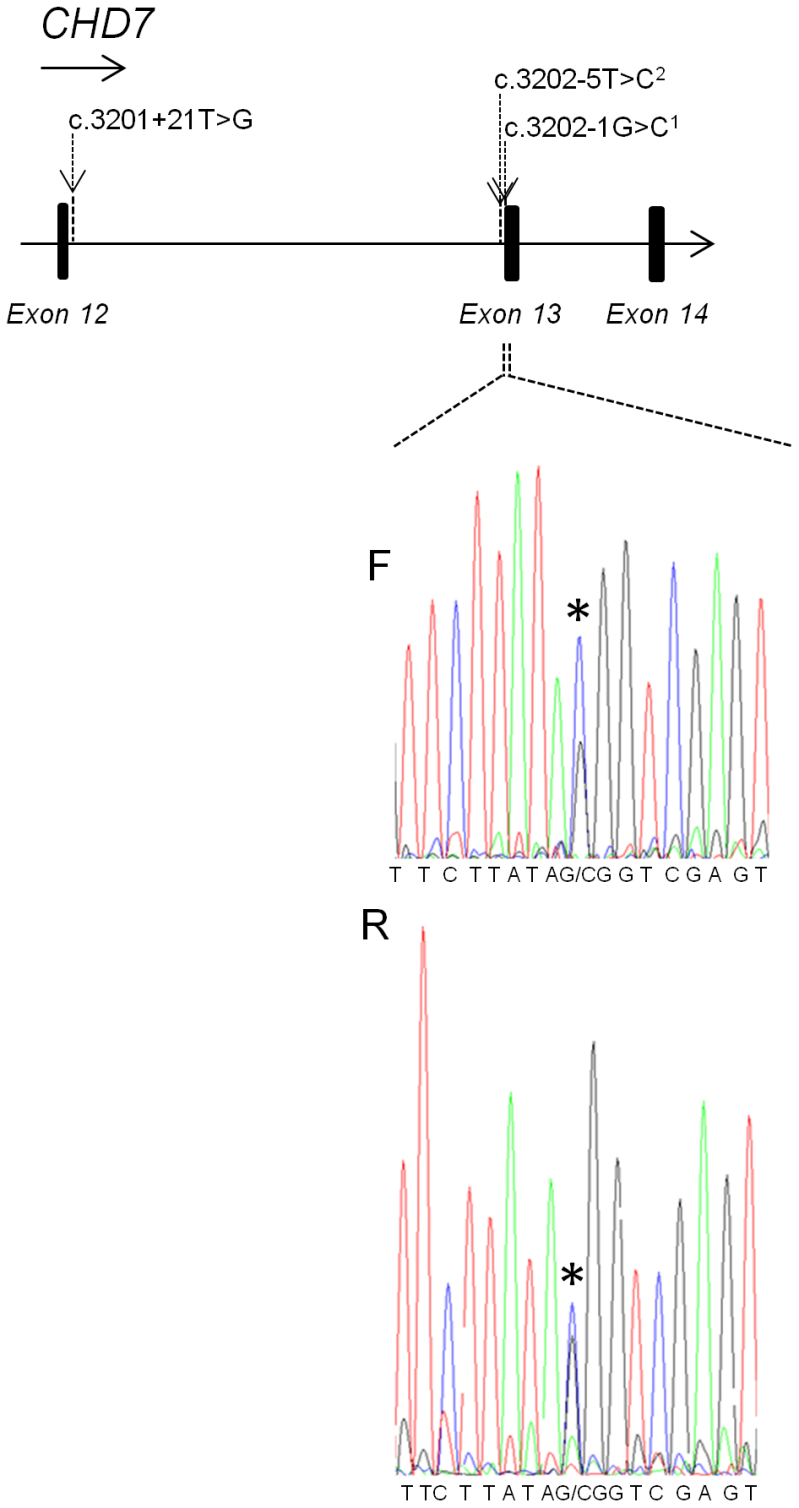
Results from DNA sequencing of the *CHD7* gene. Upper part showing single base mutations in intron 12 of the *CHD7* gene reported in the *CHD7* database (www.chd7.org) together with the pathogenic c.3202-1 G>C mutation identified in patient V15 (1). The c.3202-5T>C mutation (2) has been reported in four patients in the *CHD7* database and was found in patient V7 in our study. We consider the mutation to be of unclear clinical significance since experimental evidence does not indicate that it affects splicing. Lower part showing the pathogenic mutation c.3202-1G>C (*) detected in patient V15 in forward (F) and reverse (R) sequences.

A heterozygous *de novo* mutation (c.3202-1G>C) predicted by the Alamut program to affect splicing, was identified in patient V15 (Fig. 4, Table 4), consistent with CHARGE syndrome. The clinical manifestations of the patient include a combined heart malformation, esophageal atresia, cleft lip and palate, deafness of the left ear and micropenis. Further, a heterozygous intronic unknown variant, (c.3202-5T>C) was detected in patient V7 who has five of the VACTERL criteria and only one of the minor CHARGE criteria (Table 4). This mutation has previously been reported as pathogenic in four cases in the *CHD7* database (www.chd7.org). Of the corresponding publications [23], [24], one article is not written in English and the other does not include phenotypic descriptions or a motivation to the interpretation of the mutation as pathogenic. In our patient, DNA from the biological parents was unavailable and inheritance status could not be determined. cDNA synthesis and subsequent PCR-based amplification revealed single bands of equal size and intensity in the patient and in a healthy control when analyzed on agarose gel electrophoresis (data not shown) and sequencing of the PCR-product did not indicate that the mutation would affect *CHD7* splicing. The splice prediction function in the Alamut v2.0 software did not predict the mutation to strongly affect splicing.

### Protein-protein interaction database search

Searches in the PINA database to firstly identify possible interactions between proteins encoded by genes in the 9p-deletion and 18q-duplication, and secondly between proteins encoded by genes in the detected gene dose alterations (the 9p-deletion/18q-duplication, FANCB deletion and the gene dose alterations of unclear clinical significance) and VACTERL candidate genes, resulted in a number of putative interactions presented in Table S3.

## Discussion

We have performed array-CGH on DNA from 39 patients and fetal cases with VACTERL diagnosis or a VACTERL-like phenotype. In two out of the 19 fetal cases pathogenic gene dose alterations were identified. In one fetal case, a terminal 18q-duplication and 9p-deletion was shown to be caused by a (9;18)(p24.1;q12.3) unbalanced translocation inherited from the father who was a balanced carrier. A clinical overlap between the VACTERL and trisomy 18 phenotypes can be observed [25] and a del(9)(p23p24) has previously been described in a CHARGE patient, indicating phenotypical similarities between these conditions, although the reported patient also had a 13q-duplication [26]. In the second fetal case, a whole-gene deletion of *FANCB* was identified together with a dup(16)(p12.3p13.11) that has been associated with malformations [27], [28]. Fanconi anemia is a syndrome characterized by high risk of bone marrow failure and malignancy, and malformations in about 60% of patients [29]. Whole *FANCB* gene deletions in males with Fanconi anemia presenting as VACTERL have been reported twice, in both cases the aberrations were detected by array-CGH screening [30], [31]. It is not possible to draw any conclusion whether the 16p-duplication may have contributed to the fetal phenotype since the *FANCB* deletion alone with certainty can explain the fetal malformations. In summary our results support published data that clearly pathogenic gene dose imbalances are rarely found in VACTERL patients [10], [16], [17].

Mutation screening of PCSK5 and HOXD13 was initiated after reported mutations in VACTERL cases [18], [19], while CHD7 was screened due to the clinical overlap between CHARGE and VACTERL phenotypes. Our findings show the value of considering CHD7 analysis in patients with a VACTERL phenotype. Screening of HOXD13 and PCSK5 did not reveal any obvious pathogenic mutations and their role in the etiology of VACTERL remains unclear, however, it is possible that the detected PCSK5 variants could represent pathogenic variants with reduced penetrance. For further investigation of genes implicated in VACTERL association, it would be of interest to perform mutation screening of ZIC3, a gene associated with X-linked heterotaxy in which mutations have been detected in a few patients with VACTERL association phenotypes [13], [32].

The apparent low number of findings in genetic studies of VACTERL may be the result of “true” VACTERL cases (without additional malformations, growth restriction or facial dysmorphism) being due to other than genetic causes such as epigenetic factors, disruptive processes during pregnancy or maternal disease. Recent twin studies support a low proportion of genetic abnormalities found in VACTERL patients [33], [34].

The results of our study show, that array-CGH may be considered as a diagnostic tool in patients with VACTERL association. To search for additional causative mutations we plan systematic high throughput-sequencing in VACTERL patients.

## Supporting Information

Table S1 **Summary of clinical findings in patients and fetal cases with VACTERL association or a VACTERL-like phenotype**
(DOC)

Table S2 **Summary of genetic analyses performed in patients and fetal cases**
(DOC)

Table S3 Protein-protein interactions between proteins encoded by genes in detected CNVs and VACTERL candidate genes*
(DOC)

Table S4 **Copy number variants of unclear clinical significance detected by array-CGH**
(XLS)
