# Supplementary material for: Mutation Screening and Array Comparative Genomic Hybridization Using a 180K Oligonucleotide Array in VACTERL Association
Source: PLoS One. 2014 Jan 9;9(1):e85313. doi: 10.1371/journal.pone.0085313 (PMC3887047; doi:10.1371/journal.pone.0085313)
Supplement: Table S1 — Summary of clinical findings in patients and fetal cases with VACTERL association or a VACTERL-like phenotype (DOC) [file pone.0085313.s001.doc]

| **Table S1. Summary of clinical findings in patients and fetal cases with VACTERL association or a VACTERL-like phenotype** | | | | | | | | |
| --- | --- | --- | --- | --- | --- | --- | --- | --- |
| **Patients** | **Sex** | **Vertebral  anomalies** | **Anal atresia** | **Cardiac anomalies** | **TEF/**  **EA** | **Renal  anomalies** | **Limb anomalies** | **Other  anomalies** |
| **V1** | F |  | yes† |  |  | multicystic dysplasia | lower extremity hypoplasia | Arnold-Chiari malformation, uterine agenesis |
| **V2** | M | lumbosacral vertebral defect, scoliosis | yes |  |  | renal agenesis (right), vesicouretheral reflux |  |  |
| **V3** | M | vertebral defects LIII-LV, scoliosis | yes | pulmonary stenosis, VSD | yes |  |  | duodenal atresia |
| **V4** | M |  | yes |  | yes | right ureteric stenosis, small right kidney with impaired function |  |  |
| **V5** | M | yes | yes | VSD |  | yes | radial and thumb aplasia (right) |  |
| **V6** | F |  | yes | ASD, two VSDs, aortic hypoplasia | yes |  | radial hypoplasia, absence of right thumb | auricular tags, optic atrophy |
| **V7** | M | caudal regression syndrome | yes | ASD^*^ |  | unilateral renal agenesis (left) | lower right and upper left extremity hypoplasia | penoscrotal hypospadias |
| **V8** | M | yes |  |  | yes | horseshoe kidney |  |  |
| **V9** | M | sacral defect with hemivertebrae L5-S1 | yes | VSD and ASD |  |  |  |  |
| **V10** | M |  |  | right-sided aortic arch | yes |  |  | penile hypospadias, cryptorchidism |
| **V11** | F | hemivertebra Th10, 1,5 vertebra Th11 |  | tetralogy of Fallot with pulmonary stenosis, VSD double aortic arch | yes | no |  |  |
| **V12** | M | hemivertebra Th5, distal sacral agenesis | yes |  |  |  | yes | urinary bladder dysfunction |
| **V13** | F |  | yes |  | yes | unilateral renal agenesis (left) |  | duodenal atresia, uterus didelphys with double vagina |
| **V14** | F | vertebral malformations and scoliosis |  |  | yes |  | small thumb | single umbilical artery |
| **V15** | M |  |  | ASD, VSD, mitral and aortic valve insufficiency, right-sided aortic arch | yes |  |  | cleft lip/palate, deafness left ear, short clavicles, micropenis, cryptorchidism |
| **V16** | M |  |  | tetralogy of Fallot | yes | polycystic kidney (right) |  | omphalocele, Meckel diverticulum |
| **V17** | M |  | yes |  | yes |  |  | urethral stricture |
| **V18** | F |  | yes |  | yes | unilateral small kidney (left) |  |  |
| **V19** | F |  |  | right-sided aortic arch | yes |  |  |  |
| **V20** | F |  |  |  | yes | renal duplication (right) |  |  |
| **FC1** | F |  | yes† | complex Eisenmenger-like malformation |  | unilateral renal agenesis (left) |  | acrocephaly, dysmorphic facial features, 13 rib pairs, abnormal intestinal rotation |
| **FC2** | F |  |  | VSD, central artery and vein anomalies | yes | bilateral hydroureters with left hydronephrosis | radial agenesis with right thumb hypoplasia | brachycephaly, dysmorphic facial features, low-set ears, abnormal intestinal rotation, accessory spleens |
| **FC3** | M |  | yes | complex Fallot-like malformation, bicuspid aortic valve |  | unilateral renal agenesis (left) |  | enlarged OFC, dysmorphic facial features, low-set ears, small omphalocele, accessory spleen, single umbilical artery |
| **FC4** | F |  | yes | VSD |  | unilateral renal agenesis (left) |  | dysmorphism, hypoplastic 12^th^ rib pair |
| **FC5** | M |  |  | ASD, VSD, right ventricle hypertrophy, pulmonary ositum stenosis | yes | multicystic kidneys, hydronephrosis and hydroureters, distended urinary bladder | radial aplasia and thumb agenesis bilaterally, syndactyly of toes bilaterally | brachycephaly, dysmorphic facial features, malrotation of intestine, deformed liver, gallbladder agenesis, pyloric stenosis, generalized hydrops fetalis |
| **FC6** | F |  |  | ASD, VSD, pulmonary trunk and ostium stenosis, vena cava anomaly | yes |  |  | brachycephaly, dysmorphic facial features, cervical myelomeningocele, cleft palate, accessory spleens |
| **FC7** | F | hemivertebrae | yes | VSD, right ventricle hypoplasia, hypoplastic truncus pulmonalis | yes | fused kidneys, right ureter hypoplasia | bilateral radial hypoplasia, right thumb agenesis, left thumb hypoplasia | dysmorphic facial features, pyloric stenosis, uterine hypoplasia, thymus anomaly |
| **FC8** | M | severe thoracal scoliosis |  | VSD and bicuspid pulmonary valve |  | unilateral renal agenesis (right) |  | dysmorphic facial features, ear malformation, microphtalmia, cleft lip palate, occipital encephalocele |
| **FC9** | M |  | yes | ASD |  | horseshoe multicystic kidney |  | dysmorphic facial features, duodenal atresia |
| **FC10** | M |  | yes | complex Fallot-like malformation, absent ductus arteriosus, right-sided aortic arch |  | horseshoe kidney | rudimentary thumbs bilaterally | left ear microtia with atresia of the external auditory canal, low-set right ear |
| **FC11** | M | kyphoscoliosis | yes | complex malformation with left ventricle hypoplasia | yes | multicystic kidneys with hydronephrosis, distended urinary bladder, urethra stenosis | finger anomalies bilaterally, three toes absent on right foot | dysmorphic facial features, low-set ears, duodenal atresia, hypoplastic external genitalia, uterus not identified, single umbilical artery, 11 ribs bilaterally, cervical ribs |
| **FC12** | M |  | yes |  | yes | large multicystic kidneys |  | dysmorphic facial features, low-set ears, hypospadias, ectopic pancreas, accessory spleen, rocker bottom feet, thorax asymmetry |
| **FC13** | F |  | yes | VSD, hypoplastic truncus pulmonalis and ductus arteriosus | yes | bilateral renal and ureteric agenesis |  | brachycephaly, dysmorphic facial features, low-set ears, external genitalia not identified, uterine agenesis, agenesis of the vermiform appendix, Meckel diverticulum, hydrocephalus, single umbilical artery |
| **FC14** | M |  |  | complex malformation with VSD, right ventricle hypoplasia and outflow tract anomaly, absence of ductus arteriosus, vena cava anomaly | yes | unilateral mild hydronephrosis and hydroureter (left) |  | dilatation of 4^th^ cerebral ventricle |
| **FC15** | M | severe kyphoscoliosis | yes | right cardiac position, rectangular shape, central venous anomalies |  | cystic renal dysplasia, unilateral hydronephrosis (right) |  | dysmorphic facial features, 6 left ribs, 11 right ribs, sternum anomaly |
| **FC16** | M |  | yes | VSD |  | multicystic kidneys | absent radius and thumb (right) | brachycephaly, dysmorphic facial features, low-set ears, rocker bottom heels, single umbilical artery |
| **FC17** | F |  | yes | truncus arteriosus and VSD |  | bilateral renal agenesis | bilateral radial aplasia and absence of thumbs, left foot polydactyly | dysmorphic facial features, posteriorly angulated low-set ears, ambiguous genitalia |
| **FC18** | M |  | yes |  | yes | bilateral renal agenesis |  | low-set ears, omphalocele, left diaphragmatic hernia, single umbilical artery |
| **FC19** | F |  | yes | VSD | yes | horseshoe kidney |  | dysmorphic facial features, low-set ears, 11 ribs bilaterally |

*spontaneous closure †cloacal/cloacal-like malformation, TEF tracheoesophageal fistula, EA esophageal atresia, V fulfilling VACTERL criteria, FC fetal case, ASD atrial septal defect, VSD ventricular septal defect, OFC occipital-frontal circumference
