# Supplementary material for: Mutation Screening and Array Comparative Genomic Hybridization Using a 180K Oligonucleotide Array in VACTERL Association
Source: PLoS One. 2014 Jan 9;9(1):e85313. doi: 10.1371/journal.pone.0085313 (PMC3887047; doi:10.1371/journal.pone.0085313)
Supplement: Table S2 — Summary of genetic analyses performed in patients and fetal cases (DOC) [file pone.0085313.s002.doc]

| Table S2. Summary of genetic analyses performed in patients and fetal cases | | | | | |
| --- | --- | --- | --- | --- | --- |
| Patient/fetal case | Tissue used for DNA extraction | Array-CGH (OGT 180K) | *PCSK5* | *HOXD13* | *CHD7* |
| V1 | blood | X | X | - | - |
| V2 | blood | X | X | X | X |
| V3 | blood | X | X | X | X |
| V4 | blood | X | X | X | - |
| V5 | intestine | X | X | X | X |
| V6 | blood | X | X | X | X |
| V7 | blood | X | X | X | X |
| V8 | blood | X | X | X | X |
| V9 | blood | X | X | X | X |
| V10 | blood | X | X | - | - |
| V11 | blood | X | X | X | X |
| V12 | blood | X | X | X | X |
| V13 | blood | X | X | X | X |
| V14 | blood | X | X | X | X |
| V15 | blood | BAC 38K | X | X | - |
| V16 | blood | X | X | X | X |
| V17 | blood | X | X | X | X |
| V18 | blood | BAC 38K | X | X | - |
| V19 | blood | BAC 38K | X | X | - |
| V20 | blood | X | X | X | X |
| FC1 | liver | X | X | X | X |
| FC2 | heart | X | X | X | X |
| FC3 | spleen | X | X | X | X |
| FC4 | heart | X | X | X | - |
| FC5 | liver, lung | X | X | X | X |
| FC6 | lung | X | X | X | X |
| FC7 | spleen | X | X | X | X |
| FC8 | heart | X | X | X | X |
| FC9 | lung | X | X | X | - |
| FC10 | liver | X | X | X | X |
| FC11 | liver | X | X | X | X |
| FC12 | heart | X | X | - | - |
| FC13 | heart | X | X | - | - |
| FC14 | spleen | X | X | - | - |
| FC15 | lung | X | X | - | - |
| FC16 | heart | X | X | - | - |
| FC17 | liver | X | X | - | - |
| FC18 | heart | X | X | - | - |
| FC19 | heart | X | X | - | - |

- not analysed
