# Supplementary material for: Mutation Screening and Array Comparative Genomic Hybridization Using a 180K Oligonucleotide Array in VACTERL Association
Source: PLoS One. 2014 Jan 9;9(1):e85313. doi: 10.1371/journal.pone.0085313 (PMC3887047; doi:10.1371/journal.pone.0085313)
Supplement: Table S3 — Protein-protein interactions between proteins encoded by genes in detected CNVs and VACTERL candidate genes* (DOC) [file pone.0085313.s003.doc]

| Supplementary table 3. Protein-protein interactions according to PINA* searches | | | |
| --- | --- | --- | --- |
| Patients | Gene dose alterations | Proteins encoded by | Findings |
| FC14 | Interactions between proteins encoded by genes in del(9)(p24.3p24.1) and dup(18)(q12.3q23) | *BCL2* | *PMAIP1* |
|  |  | *CNDP1* | *CNDP2* |
|  |  | *SMAD4* | *SMAD2* |
|  |  | *SMAD2* | *NEDD4L* |
|  |  | *PIAS2* | *MBD1* |
|  |  | *SMARCA2* | *TCF4* |
|  |  | *DOCK8* | *SMAD2* |
|  |  | *RANBP6* | *SMAD2* |
|  |  | *JAK2* | *TCF4* |
|  |  | *UHRF2* | *SMAD4* |
|  | Interaction between proteins encoded by genes in del(9)(p24.3p24.1) and dup(18)(q12.3q23) and VACTERL candidate genes† | *PIAS2* | *MYCN* |
|  |  | *TCEB3C* | *PTEN* |
|  |  | *SMAD4* | *GLI3* |
|  |  | *SMAD2* | *GLI3* |
|  |  | *SMAD2* | *HOXD13* |
|  |  | *TCF4* | *FANCD1* |
|  |  | *UHRF2* | *PTEN* |
|  |  | *SERPINB5* | *FANCP* |
|  |  | *SERPINB4* | *FANCP* |
|  |  | *SALL3* | *CHD7* |
| FC10 | Interactions between proteins encoded by genes in dup(16)(p13.11p12.3) and *FANCB* | None detected | |
| Detected CNVs of unclear clinical significance in a proportion of cases (16 patients) | Interactions between CNVs of unclear clinical significance and VACTERL candidate genes† | *ANAPC3* | *PTEN* |
|  |  | *MYH11* | *FANCP* |

* PINA: Protein Interaction Network Analysis Platform database (<http://cbg.garvan.unsw.edu.au/pina/>).
† The VACTERL candidate genes were chosen by performing a search for VACTERL in the OMIM database (<http://omim.org/>) and the resulting list of genes was supplemented to encompass all *FANC* genes and the *CHD7* gene: *FANCA, FANCB, FANCC, FANCD1, FANCD2, FANCE, FANCF, FANCG, FANCI, FANCL, FANCM, FANCN, FANCO, FANCP, PTEN, HOXD13, PCSK5, SALL1, VANGL1, OFC11, SHH, MYCN, GLI3, ZIC3, MTTL1, CHD7.*
